# A Retrospective Analysis of the Efficacy of Immunotherapy in Metastatic Soft-Tissue Sarcomas

**DOI:** 10.3390/cancers12071873

**Published:** 2020-07-11

**Authors:** Varun Monga, Keith M. Skubitz, Seth Maliske, Sarah L. Mott, Hilary Dietz, Angela C. Hirbe, Brian A. Van Tine, Peter Oppelt, Scott Okuno, Steven Robinson, Madeline O’Connor, Mahesh Seetharam, Steven Attia, John Charlson, Mark Agulnik, Mohammed Milhem

**Affiliations:** 1 Division of Hematology, Oncology, and Blood and Marrow Transplantation, University of Iowa Carver College of Medicine, Iowa City, IA 52242, USA; seth-maliske@uiowa.edu (S.M.); mohammed-milhem@uiowa.edu (M.M.); 2 Division of Hematology and Oncology, University of Minnesota Medical School, Minneapolis, MN 55455, USA; skubi001@umn.edu; 3 Holden Comprehensive Cancer Center, University of Iowa, Iowa City, IA 52242, USA; sarah-mott@uiowa.edu; 4 Division of Hematology, Oncology, and Blood and Marrow Transplantation, Washington University, St. Louis, MO 63130, USA; hdietz@wustl.edu (H.D.); hirbea@wustl.edu (A.C.H.); bvantine@wustl.edu (B.A.V.T.); poppelt@wustl.edu (P.O.); 5 Division of Medical Oncology, Mayo Clinic, Rochester, MN 55902, USA; okuno.scott@mayo.edu (S.O.); Robinson.Steven@mayo.edu (S.R.); madelineoconnor97@gmail.com (M.O.); 6 Division of Hematology and Medical Oncology, Mayo Clinic, Phoenix, AZ 85058, USA; Seetharam.Mahesh@mayo.edu; 7 Division of Hematology and Medical Oncology, Mayo Clinic, Jacksonville, FL 32224, USA; Attia.Steven@mayo.edu; 8 Division of Hematology, Oncology, and Blood and Marrow Transplantation, Medical College of Wisconsin, Milwaukee, WI 53226, USA; jcharlso@mcw.edu; 9 Division of Hematology, Oncology, and Blood and Marrow Transplantation, Northwestern University, Chicago, IL 60611, USA; Mark.Agulnik@nmff.org

**Keywords:** immunotherapy, sarcoma, efficacy, anti-PD1, anti CTLA-4

## Abstract

Although checkpoint inhibitors have been approved in multiple cancers, they are still under investigation in soft tissue sarcoma (STS). We conducted a retrospective review to report the safety, efficacy, and prognostic factors related to checkpoint inhibitors in STS. A sequential cohort of metastatic STS patients from four institutions treated with checkpoint inhibitors was assembled. Logistic and Cox regression models were applied to determine the effect of patient characteristics, prior treatment, and baseline factors on achieving the best overall response of complete response (CR), partial response (PR), or stable disease (SD) as determined by the treating physician. Eighty-eight patients with two median prior therapies received checkpoint inhibitors. Treatments included pembrolizumab in 47, nivolumab in 6, ipilimumab in 1, combination ipilimumab/nivolumab in 27, and other combination immunotherapies in 7 patients. Immunotherapy was discontinued in 54 patients—72.2% for progression, 16.7% for toxicity, and 11.1% for other reasons. Median progression-free survival (PFS) was 4.1 months and median overall survival was 19.1 months. One patient with undifferentiated pleomorphic sarcoma (UPS) achieved a CR, while 20 patients had a PR, including 7 UPS, 9 leiomyosarcoma (LMS), and 1 each with alveolar soft part sarcoma, fibroblastic sarcoma, sclerosing epithelioid fibrosarcoma, and myxofibrosarcoma. Forty-five percent (9 of 20) of LMS patients achieved a PR. Twenty-eight patients had SD. Our results confirm the activity and safety of anti-PD-1 therapy in metastatic STS. A notable response rate was observed in UPS and LMS subtypes. This study expands the knowledge base beyond what is currently available from clinical trials involving checkpoint inhibitors in metastatic STS.

## 1. Introduction

Sarcomas include a heterogeneous group of rare, malignant solid tumors of mesenchymal origin that constitute approximately 1% of all adult malignancies, 15% of pediatric malignancies and consist of more than 100 different subtypes [1,2]. Surgery remains the mainstay of therapy for localized disease. Radiation and chemotherapy have roles in neo-adjuvant and adjuvant settings, as well as metastatic disease. Given their rarity and diversity, treatment for metastatic soft tissue sarcoma (STS) remains challenging, as does enrollment in clinical trials. Doxorubicin, either alone or in combination with ifosfamide, is the standard of care for most patients [1,3]. Other chemotherapeutic or targeted agents FDA-approved for use in management of STS include pazopanib, ifosfamide, trabectedin, gemcitabine, docetaxel, and dacarbazine. Nevertheless, metastatic disease has a very poor prognosis with current agents.

Programmed death 1 (PD-1) and cytotoxic T-lymphocyte-associated antigen 4 (CTLA-4) are surface receptors expressed on T cells. Upon binding to their respective ligands on tumor cells, they function to downregulate the host immune response by interfering with cytokine secretion and lytic activity. Targeting this inhibitory axis via monoclonal antibodies against PD-1, PD-L1, and CTLA-4 has been termed checkpoint inhibition, and has shown remarkable success in multiple malignancies [4].

Immune checkpoint inhibitors (IPI) have also been recently explored in sarcomas. In the phase 2 SARC 28 trial, pembrolizumab, an anti-PD-1 antibody, was used as a single agent in relapsed refractory soft tissue and bone sarcomas at 12 academic centers and showed an 18% overall response rate (ORR) (95% CI, 7–33) [5]. The response was most noticeable in undifferentiated pleomorphic sarcoma (UPS) subtype (ORR 40%). Additional STS subtypes demonstrating a response included liposarcoma (ORR 20%) and synovial sarcoma (10%). In a multicenter, open-label, non-comparative, randomized, phase 2 study, nivolumab, another anti-PD1 agent, when used in combination with ipilimumab, an anti-CTLA-4 Ab, reported response rates of 16% (92% CI, 7–30%) compared to 5% with nivolumab alone [6]. Authors from both studies concluded that single-agent IPI and combination IPI have promising efficacy in certain sarcoma subtypes.

Although prior studies have demonstrated activity, due to the disease rarity, data supporting use of IPI is limited for many other sarcoma subtypes. Because patients were treated with these agents off-label as part of clinical care when the drugs became available, a significant amount of real-world data exists in this setting compared with the number of patients who have been enrolled and treated in immunotherapy clinical trials. We performed a retrospective chart review of patients with STS treated with these agents from four Midwest Sarcoma Trials Partnership institutions (University of Iowa, University of Minnesota, Mayo Clinic, Washington University St. Louis). Treatments were part of a clinical trial with published results or as off-label use to better define toxicities, response rates and PFS in the various sarcoma subtypes. We also analyzed patient demographics and clinical data to better identify clinical variables that may predict improved outcomes.

## 2. Results

Patient demographics and baseline characteristics are summarized in Table 1. Overall, 88 patients with a median age of 55 years (range 11–80) were included for review. IPIs were used in the frontline setting in 14 patients (15.9%). Patients had received a median 2 prior therapies (range 1–8). IPI use consisted of pembrolizumab in 47, nivolumab in 6 and ipilimumab in 1, combination ipilimumab/nivolumab in 27, and other combinations of immunotherapy in 7 patients. Other combinations consisted of cabiralizumab plus nivolumab in 1 patient and 66 patients receiving non-standard dosing of the other three IPIs. Off-label concurrent chemotherapy was given in 5 patients (5.7%), while radiation was offered concurrently in 16 patients (18.2%). Concurrent chemo-immunotherapy was used off-label to treat angiosarcoma in 2, UPS in 1, LMS in 1, and epithelioid sarcoma (ES) in 1. Median follow-up was 11.4 months. At the time of analysis, immunotherapy was discontinued in 54 patients—72.2% for progression and 16.7% for toxicity. Immune-mediated toxicity occurred in 21 patients, with a majority needing treatment for adverse events (AE; 81.0%). Twelve patients (31.6%) receiving pembrolizumab and 5 patients (18.5%) receiving combination ipilimumab and nivolumab developed an immune-mediated AE. We did not characterize the grade of toxicity.

**Figure 1:**
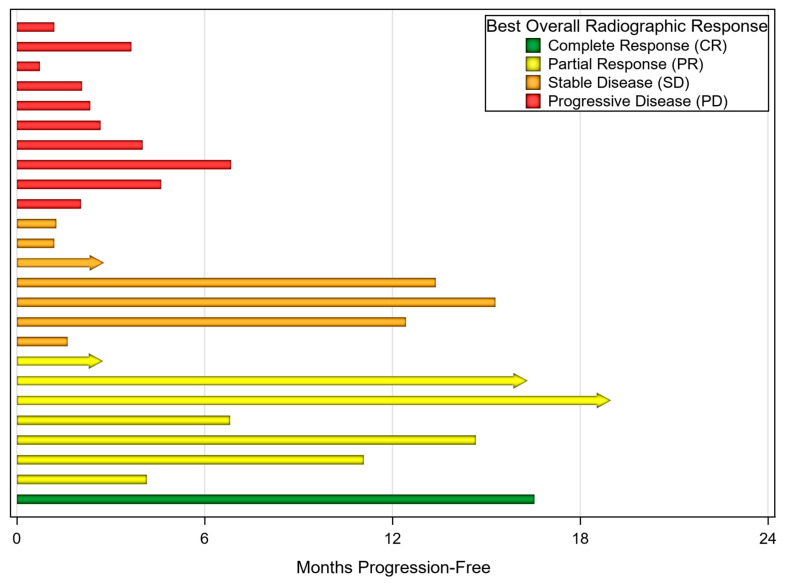
Month progression-free grouped by best overall radiographic response in patients with undifferentiated pleomorphic sarcoma (UPS) (*n* = 25). Arrows indicate ongoing immunotherapy treatment at the time of analysis. One patient continued treatment beyond first progression at 6 months.

The ORR for the entire cohort was 23.9% (21 of 88). One patient with UPS achieved complete response (CR), while 20 other patients had a PR, including seven UPS, nine LMS, and one each of alveolar soft part sarcoma, fibroblastic sarcoma, sclerosing epithelioid fibrosarcoma, and myxofibrosarcoma. Nine of 20 LMS patients (45.0%) achieved a PR. Twenty-eight patients (31.8%) had stable disease as their best clinical response. Of the 21 patients with ORR, 13 have progressed with a median time to progression of 14.4 months. Clinical benefit was seen in 46.8% of patients receiving pembrolizumab alone compared to 70.4% of those receiving ipilimumab and nivolumab. One patient achieved a CR with pembrolizumab. A PR was achieved in 17.0% of those receiving pembrolizumab and 37.0% of those receiving ipilimumab and nivolumab. No patients receiving nivolumab alone responded, but 4 of 6 patients had stable disease. Median PFS was 4.1 months and median overall survival was 19.1 months. Best overall clinical response of the 25 UPS patients can be seen in Figure 1.

On univariate analysis gender, prior treatments, age, neutrophil:lymphocyte ratio, the number of prior lines of treatment, and the type of prior treatment were not associated with response or PFS (Table 1).

## 3. Discussion

Patients with metastatic STS have a poor prognosis with few effective options for systemic therapy. The diversity and rarity of this heterogeneous group of diseases makes it more difficult to study. Despite these limitations, immunotherapy is emerging with encouraging results. Immunotherapeutic options in sarcoma have been studied, including oncolytic viruses and vaccine therapy [7,8]. More recently, IPI have been studied, with variable success, leading to their selected use in undifferentiated pleomorphic sarcomas [1]. More recently, IPI have been used as an off-label therapy across numerous STS subtypes.

Early studies assessing the benefit of checkpoint inhibitors in STS showed conflicting results. Two single-center studies showed no ORR (0%) in 18 enrolled patients, 12 uterine-LMS patients and 6 synovial sarcomas [9,10]. In the SARC028 multicenter phase II study, pembrolizumab monotherapy was administered to patients with STS and bone sarcomas. In 40 patients with STS, the overall response was 18% (95% CI, 7–33) and 12-week PFS was 55% (95% CI, 42–71). STS-subtypes included were LMS, synovial sarcoma (SS), liposarcoma (LpS), and UPS, with ORR of 0%, 10%, 20%, and 40%, respectively. There was one CR noted in the UPS subtype [5]. In another study, nivolumab as a single agent was retrospectively studied in 24 patients with STS and bone sarcoma, revealing an overall response of 12.5% (3 of 24), while 50% (12 of 24) achieved clinical benefit (PR + SD). Of those with STS histologies, only 1 of 18 patients achieved ORR (PR with ES, 5.6%). Specific STS histologies included in this retrospective study were ES, rhabdomyosarcoma (RMS), malignant peripheral nerve sheath tumor (MPNST), UPS, desmoplastic small round cell tumor, synovial sarcoma, LMS, intimal sarcoma, and LpS [11]. In the Alliance A091401 phase II non-comparative study, 76 total patients with STS (LMS, LpS, spindle cell sarcoma, SS, myxofibrosarcoma, angiosarcoma, and MPNST) received either nivolumab monotherapy at 3 mg/kg every 2 weeks or ipilimumab (1 mg/kg) in combination with nivolumab (3 mg/kg) every 3 weeks. A higher ORR was observed in the combination therapy group (6 of 38, 16% in LMS, myxofibrosarcoma, UPS, and angiosarcoma, 92% CI, 7–30%) compared to nivolumab monotherapy (2 of 38, 5% in alveolar soft part sarcoma and LMS, 92% CI, 1–16%). In this study, promising responses were again seen with UPS, with a PR seen in two of six patients treated with combination IPI [6]. However, in the PEMBROSARC trial in which patients received metronomic dosing of cyclophosphamide 50 mg twice daily, 1 week on and 1 week off, with pembrolizumab 200 mg every 3 weeks, no patients with UPS or LMS showed a response [12].

Our cohort of 88 patients showed similar responses with the anti-PD1 agents. Nivolumab monotherapy did not induce a response in any of six patients treated. In comparison, single-agent pembrolizumab elicited an overall response in nine of 47 (19.1%) patients, including 1 complete response, and combination nivolumab/ipilimumab showed an overall response in 10 of 27 (37.0%) patients. Furthermore, 8 of 25 patients (32%) with UPS had ORR, with one patient achieving a CR. Similarly, those with LMS showed activity with IPI therapy, as nine of 20 patients (45.0%) with LMS achieved a PR. In the remaining subtypes, objective responses were rare, with four patients (6.9%) achieving a PR. This included one each of alveolar soft part sarcoma, fibroblastic sarcoma, epithelioid fibrosarcoma, and myxofibrosarcoma. Our data support that, at least for certain sarcoma subtypes, single-agent pembrolizumab and combination immunotherapy are both able to evoke an anti-tumor effect.

Predicting which sarcoma subtypes garner the greatest benefit from these therapies remains to be defined. Our data and data from others suggest that specific subtypes of sarcoma may predict the response to immunotherapy. UPS appears to be a subtype with a favorable response to IPI. As previously mentioned, SARC028 showed a more pronounced benefit in UPS as the ORR was 40% with one CR and three PRs of 10 evaluable patients. In our cohort of 101 patients, eight of 25 patients (32.0%) with UPS had ORR, with one patient achieving a CR. Upon univariate analysis, UPS did not predict for improved odds of achieving a clinically meaningful benefit, possibly due to the small sample size. Similarly, UPS did not show improved PFS compared to those with other histologies. Although the odds of achieving a clinical benefit in UPS compared with the other histological subtypes did not appear to be statistically significant, a clinical benefit rate of 32% is valuable and should be explored further in future randomized trials.

LMS may be another subtype in which IPI have activity. In our cohort, 45.0% (nine of 20) patients with LMS achieved a PR. The primary anatomic sites of LMS in these patients were: intrabdominal (3), retroperitoneal (1), extremity (3) and uterine (2). Prior studies have demonstrated activity, but they have not shown this degree of response [5,6]. One possible reason for this could be the elevated tumor mutation burden after the failure of multiple lines of prior chemotherapies and or radiation leading to a better response to IPI as shown in some other tumor types [2,13]. Of note, two out of 20 patients had radiation-induced LMS, of which one had a PR to immunotherapy. We did not investigate PD-L1 status, tumor mutational burden nor microsatellite instability status within the tumors, which is a limitation. This would be the focus of further studies. It would be important to study IPI in LMS in future randomized clinical trials with special attention to biomarkers that may help predict the response.

It is not clear why UPS and LMS appear sensitive to IPI. In other malignancies, intra-tumoral PD-L1 positivity by immunohistochemical staining (IHC) has been shown to predict response rates with positive staining tumors having a doubled response rate compared to tumors showing no staining [14]. The immunogenic potential of sarcomas has been debated. Some studies have suggested that sarcomas have limited immunogenic potential. In the SARC028 trial, only 4% of pretreatment tumors stained positive for PD-L1 (>1%) [5]. D’Angelo et al. reported that 12% of STS stained positive for PD-L1; however, a majority of these were gastrointestinal stromal tumors (GISTs) [15]. In that cohort, only 5.6% (2 of 36) of the other tumor samples stained for PD-L1, with none for LMS, RMS, and SS. In comparison, other groups have shown 50–65% of sarcomas express PD-L1 by IHC and are present with PD-1+ tumor infiltrating lymphocytes [2,16], and certain sarcomas may be more immunogenic than others [17]. UPS and LMS are known to have high expressions of antigen presentation and T-cell infiltration-related genes. UPS also demonstrate higher levels of PD-L1, PD-1, and T-cell infiltration compared to other sarcomas [18]. These studies did not define a cutoff or predicted response to immunotherapy. In our study, we did not specifically evaluate PD-L1 positivity by IHC, but this is an important avenue for future trial design. Furthermore, with mixed evidence that PD-L1 expression is a relevant biomarker in selecting sarcoma patients for immunotherapy, it is important to identify reliable markers of tumor response. Petiprez et al. recently published a study identifying a unique lymphoid structure that contains a high concentration of T cells and follicular dendritic cells and is particularly rich in B cells [19]. This gene expression profile was screened in pathology samples from the SARC028 clinical trial. Those with this immune-rich profile were associated with a high response rate to PD-1 blockade with pembrolizumab and improved overall survival. This marks a new milestone in the field of biomarkers for clinical decision making beyond PD-L1 IHC and histology-driven analysis.

The use of IPI in our cohort of sarcoma patients was met with a predictable toxicity profile. Overall, 27.6% of patients suffered from immune-specific toxicities. This toxicity caused nine patients (10%) to discontinue therapy altogether. No patients suffered fulminant or fatal toxicity with immune checkpoint blockade. This is comparable to the expected toxicity seen with immunotherapy, where 25% and 35% acquire immune toxicity associated with use of anti-PD1 and anti-CTLA4, respectively [20,21]. When these agents are used in combination, the risk for toxicity increases significantly. In the CheckMate 067 trial, grade-3/4 toxicities occurred in 59% of patients receiving combination ipilimumab and nivolumab [22]. In our cohort, 30.7% of patients were given combination CPI. As such, we could expect a higher toxicity rate; however, there are limitations in reporting toxicities outside of a clinical trial. This study reflects the real-life cohort of patients who are treated for metastatic STS independent of the clinical trial. Though some patients were on clinical trial, the majority were not. We did not investigate specific comorbidities, but patients as old as 80 years received therapy in this study, though patients were excluded if they had ECOG scores of 3–4. Up to 10% of patients had an ECOG score of 2. Although patients were older and sicker than most clinical trial patients, patients in our cohort tolerated therapy well. Therefore, our data suggest checkpoint inhibitors can be given without unexpected toxicities.

Our study was limited by both the retrospective design and relatively small cohort size. As a multi-institutional study, there was no central confirmation of either toxicities or radiographic response and as such were based on individual oncologist assessments. The small cohort size limited the ability to define a potential benefit with individual checkpoint inhibitors. The small sample size also limited the ability to evaluate for benefit in specific histological subtypes of STS. Because LMS can be poorly differentiated and has pleomorphic areas, it is fair to raise the question of the continuum with UPS. Because of the multi-institutional design, it was not feasible to review pathology for all patients with LMS to determine whether the responding patients had more poorly differentiated histologies. The multi-institutional, retrospective design also limited the ability to best characterize response by RECIST criteria and toxicities by CTCAE criteria, creating heterogeneity in this data. However, this study greatly increases the available data on the use of immunotherapy in STS. We strongly encourage the ongoing investigation of these agents in STS, specifically UPS and LMS.

## 4. Materials and Methods

Patients: Patients > 18 years of age treated from January 2013 to January 2018 at four Midwest Sarcoma Trials Partnership institutions (University of Iowa, University of Minnesota, Mayo Clinic, Washington University St. Louis) were retrospectively reviewed. Patients had biopsy-confirmed STS and received an IPI, either as off-label, compassionate use, or in a clinical trial for which results have been published. Patients had received at least one dose of anti-PD1 (pembrolizumab, nivolumab) or anti-CTLA4 (ipilimumab) therapy, either alone or in combination, or with another immunotherapy agent. Pembrolizumab was used at 200 mg IV every 3 weeks, intravenous nivolumab 3 mg/kg every 2 weeks, and for combination therapy dosing was nivolumab 3 mg/kg plus ipilimumab 1 mg/kg every 3 weeks for four doses followed by nivolumab monotherapy (3 mg/kg) every 2 weeks for up to 2 years. Ten patients treated with the combination of cabiralizumab (CSF-1R inhibitor) plus nivolumab were treated with 3 mg/kg every 3 weeks of nivolumab and dose escalation of the investigational agent as per protocol. Patients with bone sarcoma histologies (including osteosarcoma, Ewing’s sarcoma, chondrosarcoma) and with ECOG ≥ 3 were excluded from analysis. This study was approved by all respective Institutional Review Boards (IRB). Data usage agreements were implemented with the institutions contributing unidentified data.

Clinical Data: The demographics collected for all patients included gender, age, race, and ethnicity. The clinical data obtained for all patients included neutrophil:lymphocyte ratio, diagnosis, primary tumor location, metastatic sites, mortality status, time point of death, prior treatment data, date of treatments, including names of drugs and the number of cycles, date checkpoint inhibitor agent given, drugs given with checkpoint inhibitors, radiation therapy data, disease response as determined by the investigator, clinical benefit per treating physician, toxicities as reported in patient charts, date checkpoint inhibitor was discontinued, date of disease progression, and the date of death. Immune-related toxicities were further assessed, including the need for intervention. Imaging consisted of PET/CT or CT scans with IV contrast repeated every two or three months to assess for disease response by the treating physician. Patient performance status, as determined by ECOG score, was recorded when available.

Endpoints: The primary endpoints were the best overall clinical response, overall survival, and progression-free survival. The overall clinical response was defined as a complete response (CR), a partial response (PR), stable disease (SD), or progressive disease (PD) per treating oncologist. Overall survival is defined by the number of months from the start of immunotherapy to the date of death or last follow-up. Progression-free survival is defined as the number of months from the start of immunotherapy to the time of disease progression or death from any cause. Overall response rate (ORR) was defined as those achieving a PR or CR. A clinical benefit was defined as those patients with a CR, PR, or SD.

Statistical Analysis: Logistic regression models were applied to determine the effect of patient, prior treatment, and baseline factors on having the best overall response of CR, PR, or SD. Similarly, Cox regression models were used to assess the effect of patient, prior treatment, baseline, and treatment factors on progression-free survival. The estimated effects of predictors are reported as odds ratios (OR) or hazard ratios (HR) along with 95% confidence intervals. All statistical testing was two-sided and assessed for significance at the 5% level using SAS v9.4 (SAS Institute, Cary, NC, USA).

## 5. Conclusions

Our results are consistent with other studies suggesting activity of anti-PD-1 therapy in all types of STS. The effect in UPS is more pronounced, with 32.0% of patients achieving a PR or CR, and in LMS subtype, where 45.5% achieved a PR. The use of checkpoint inhibitors is safe in STS. The results of this study support the use of anti-PD-1 therapy in UPS, where safety and efficacy clearly show benefit, while their activity in less common subtypes is less clear. This study expands the knowledge base beyond what is currently available from clinical trials involving these agents in advanced STS. Clinical trials evaluating the efficacy of immunotherapy in STS, specifically UPS, are encouraged.

## Figures and Tables

**Table 1:** Patient baseline characteristics.

			Subtype			
Covariate		LEVEL	UPS N = 25	LMS N = 20	Other N = 43	Total N = 88
Site		University of Iowa	4 (16.0)	3 (15.0)	8 (18.6)	15 (17.0)
		Mayo Clinic	6 (24.0)	1 (5.0)	5 (11.6)	12 (13.6)
		University of Minnesota	10 (40.0)	2 (10.0)	9 (20.9)	21 (23.9)
		Wash U, St. Louis	5 (20.0)	14 (70.0)	21 (48.8)	40 (45.5)
Gender		Female	7 (28.0)	14 (70.0)	18 (41.9)	39 (44.3)
		Male	18 (72.0)	6 (30.0)	25 (58.1)	49 (55.7)
Race		Asian	1 (4.0)	0 (0)	0 (0)	1 (1.1)
		Black/African-American	1 (4.0)	4 (20.0)	4 (9.3)	9 (10.2)
		Hispanic or Latino	1 (4.0)	0 (0)	0 (0)	1 (1.1)
		White	22 (88.0)	16 (80.0)	39 (90.7)	77 (87.5)
		Extremity/trunk	20 (80.0)	6 (30.0)	26 (60.5)	52 (59.1)
Primary Tumor Location		Retroperitoneal	1 (4.0)	1 (5.0)	5 (11.6)	7 (8.0)
		Head and neck	1 (4.0)	0 (0)	3 (7.0)	4 (4.5)
		Abdominal/pelvic	3 (12.0)	13 (65.0)	9 (20.9)	25 (28.4)
		No	6 (24.0)	1 (5.0)	7 (16.3)	14 (15.9)
Prior treatment		Yes	19 (76.0)	19 (95.0)	36 (83.7)	74 (84.1)
		Gemcitabine-based	7	15	12	34
Prior treatment		Anthracycline-based	17	12	20	49
		TKI-based	6	8	13	27
		Investigational drug	4	12	8	24
		Trabectedin	2	12	2	16
		4+	12 (50.0)	9 (45.0)	21 (48.8)	42 (48.3)
Neutrophil/lymphocyte ratio		<4	12 (50.0)	11 (55.0)	22 (51.2)	45 (51.7)
		Missing	1	0	0	1
		Locally Advanced	1 (4.0)	0 (0)	7 (16.3)	8 (9.1)
Tumor status when starting therapy		Metastatic	24 (96.0)	20 (100)	36 (83.7)	80 (90.9)
		0	9 (36.0)	10 (50.0)	23 (53.5)	42 (47.7)
ECOG score		1	13 (52.0)	8 (40.0)	15 (34.9)	36 (40.9)
		2	3 (12.0)	2 (10.0)	5 (11.6)	10 (11.4)
		Ipilimumab	0 (0)	0 (0)	1 (2.3)	1 (1.1)
Immunotherapy treatment		Nivolumab	0 (0)	1 (5.0)	5 (11.6)	6 (6.8)
		Pembrolizumab	21 (84.0)	5 (25.0)	21 (48.8)	47 (53.4)
		Ipilimumab with Nivolumab	2 (8.0)	12 (60.0)	13 (30.2)	27 (30.7)
		Other	2 (8.0)	2 (10.0)	3 (7.0)	7 (8.0)
		No	17 (89.5)	16 (84.2)	36 (94.7)	69 (90.8)
Clinical Trial		Yes	2 (10.5)	3 (15.8)	2 (5.3)	7 (9.2)
		Missing	6	1	5	12
		No	19 (76.0)	17 (85.0)	36 (83.7)	72 (81.8)
Concurrent radiation		Yes	6 (24.0)	3 (15.0)	7 (16.3)	16 (18.2)
		No	24 (96.0)	19 (95.0)	40 (93.0)	83 (94.3)
Concurrent chemotherapy		Yes	1 (4.0)	1 (5.0)	3 (7.0)	5 (5.7)
		Complete Response (CR)	1 (4.0)	0 (0)	0 (0)	1 (1.1)
Best overall radiographic response		Partial Response (PR)	7 (28.0)	9 (45.0)	4 (9.3)	20 (22.7)
		Stable Disease (SD)	7 (28.0)	3 (15.0)	18 (41.9)	28 (31.8)
		Progressive Disease (PD)	10 (40.0)	8 (40.0)	21 (48.8)	39 (44.3)
		No	10 (40.0)	6 (30.0)	18 (41.9)	34 (38.6)
Immunotherapy stopped		Yes	15 (60.0)	14 (70.0)	25 (58.1)	54 (61.4)
		Progression	12 (80.0)	10 (71.4)	17 (68.0)	39 (72.2)
Reason for stopping immunotherapy		Toxicity	2 (13.3)	2 (14.3)	5 (20.0)	9 (16.7)
		Other	1 (6.7)	2 (14.3)	3 (12.0)	6 (11.1)
		No	13 (68.4)	12 (63.2)	30 (78.9)	55 (72.4)
Immune-mediated adverse event		Yes	6 (31.6)	7 (36.8)	8 (21.1)	21 (27.6)
		Missing	6	1	5	12
		No	3 (50.0)	1 (14.3)	0 (0)	4 (19.0)
Intervention for adverse event		Yes	3 (50.0)	6 (85.7)	8 (100)	17 (81.0)
		No	5 (20.0)	5 (25.0)	11 (25.6)	21 (23.9)
Patient with progressive disease		Yes	20 (80.0)	15 (75.0)	32 (74.4)	67 (76.1)
			25	20	43	88
Age at therapy start	N		60	64	54	59
	Median		(31–82)	(34–81)	(21–80)	(21–82)
	(Min-Max)		24	20	43	87
Most recent ANC ^a^ (cells/mm^3^)	N		4040	3150	4100	4000
	Median		(1600–33,720)	(230–9200)	(1330–28,470)	(230–33,720)
	(Min-Max)		24	20	43	87
Most recent ALC ^b^ (cells/mm^3^)	N		995	850	1000	1000
	Median		(200–2600)	(160–2100)	(100–2650)	(100–2650)
	(Min-Max)		24	20	43	87
N/L ratio	N		4.0	3.4	3.5	3.8
	Median		(0.9–32.4)	(0.3–18.0)	(0.7–80.0)	(0.3–80.0)
	(Min-Max)		19	19	36	74
Number of prior lines of treatment ^1^	N		2	3	2	2
	Median		(1–8)	(1–8)	(1–6)	(1–8)
	(Min-Max)		6	3	7	16
Radiation dose (Gy)	N		65	30	55	50
	Median		(25–100)	(10–45)	(30–300)	(10–300)
	(Min-Max)		25	20	43	88
Length of follow-up (months)	N		11.3	12.3	10.6	11.1
	Median		(1.2–24.2)	(0.9–31.3)	(0.1–31.7)	(0.1–31.7)
	(Min-Max)					

^a^ Absolute neutrophil count (ANC). ^b^ Absolute lymphocyte count (ALC). ^1^ Fourteen patients received first-line immunotherapy.
